# Growth Monitoring and Promotion Practice and Associated Factors Among Healthcare Workers in Public Health Facilities of Central Zone of Tigray, Ethiopia, 2023/2024: Mixed Study Design

**DOI:** 10.1155/bmri/1726994

**Published:** 2026-10-26

**Authors:** Gebrehiwet Haile Bezabh, Gebregziabher Kidanemariam Asfaw

**Affiliations:** ^1^ Department of Pediatrics School of Nursing Aksum University Aksum Ethiopia aku.edu.et; ^2^ Department of Midwifery Aksum University Aksum Ethiopia aku.edu.et

**Keywords:** growth monitoring, healthcare workers, promotion practice

## Abstract

**Background:**

Growth monitoring promotion (GMP) is a preventive and promotive child health activity that integrates growth monitoring with health and nutrition counseling. Appropriate GMP practice during the first 2 years of life is essential for preventing malnutrition.

**Objective:**

To assess GMP practice and associated factors among healthcare workers at public health facilities in central zone Tigray, Ethiopia, 2023/2024.

**Methods:**

An institution‐based mixed‐methods study was conducted among 407 healthcare workers. Ten woredas were selected using simple random sampling, and healthcare workers directly involved in GMP were included. Quantitative data were collected using self‐administered questionnaires, whereas qualitative data were obtained through in‐depth and key informant interviews using semistructured questions. Quantitative data were analyzed using SPSS Version 23, and statistical significance was declared at *p* < 0.05. Qualitative data were analyzed thematically.

**Results:**

The prevalence of appropriate GMP practice was 45.9% (95% CI: 40.8–50.6). Diploma‐level education (AOR = 0.260; 95% CI: 0.073–0.923), good knowledge (AOR = 3.091; 95% CI: 1.499–6.373), nursing profession (AOR = 2.489; 95% CI: 1.211–5.117), work experience of ≥ 11 years (AOR = 4.722; 95% CI: 1.00–7.42), training (AOR = 3.404; 95% CI: 1.386–8.363), and lower workload (AOR = 6.651; 95% CI: 3.647–12.129) were significantly associated with GMP practice.

**Conclusion and Recommendation:**

Level of education, profession, work experience, knowledge, training, and workload were factors associated with growth monitoring and promotion practice. The regional health bureau should strengthen training, ensure adequate human resources, and retain more experienced healthcare workers at GMP practice sites.

## 1. Introduction

Growth monitoring promotion (GMP) is a preventive and promotive child health activity that integrates growth monitoring with nutrition and health counseling. It involves periodic measurement of children′s weight, plotting measurements on growth charts, interpreting growth patterns, and providing caregivers with appropriate advice on child feeding and health. GMP is particularly important during the first 2 years of life, a critical period for physical and mental development, when early identification of growth problems can support timely interventions and prevent malnutrition [1–4]. Regular growth monitoring helps healthcare workers identify growth faltering, counsel caregivers, refer children requiring further assessment, and strengthen access to essential child health services [5, 6].

Despite its potential contribution to improving child nutritional status, the implementation of GMP remains challenging in many low‐ and middle‐income countries. Previous studies have identified gaps between the intended objectives of GMP and its actual implementation, including inadequate growth chart utilization, poor interpretation of growth patterns, and weak linkage between growth monitoring and individualized counseling [4, 7]. The World Health Organization (WHO) growth standards provide a common reference for assessing child growth and are intended to support accurate interpretation of children′s nutritional status [8].

Childhood malnutrition remains a major public health concern worldwide, particularly in sub‐Saharan Africa and Ethiopia. According to the 2019 Ethiopia Mini Demographic and Health Survey, 37% of children under five were stunted, 7% were wasted, and 21% were underweight [9]. In Tigray Region, the 2016 Ethiopian Demographic and Health Survey reported substantial levels of stunting, underweight, and wasting among children [10]. Malnutrition can negatively affect children′s physical growth, cognitive development, health, and future productivity [11, 12]. Consequently, GMP is included among strategies intended to improve child nutritional status through early detection of growth problems and caregiver education [13].

Evidence from different settings demonstrates variation in GMP practice. Studies conducted in South Africa, Ghana, Rwanda, and Ethiopia reported different levels of growth monitoring utilization and practice [5, 6, 14–18]. In Ethiopia, a study conducted in North Gondar reported GMP practice among health workers at 50.4%, whereas a study in Bahir Dar reported 30.3% [5, 6]. Furthermore, a study conducted in Tigray found that only 16.8% of health workers provided counseling to mothers or caregivers based on the child′s growth curve [19]. These findings indicate gaps in growth chart interpretation, counseling, and implementation of GMP procedures.

Several factors influence GMP practice among healthcare workers, including educational level, professional category, work experience, knowledge, training, availability of equipment, workload, and supportive supervision [3, 5–7]. Studies have reported associations between healthcare workers′ knowledge and GMP practice, with better knowledge linked to improved implementation in some settings [6, 16]. Adequate equipment, training, and supportive supervision may also facilitate the provision of GMP services, whereas high workload, inadequate supplies, and insufficient practical skills can hinder service delivery [6, 7, 20]. However, findings regarding the relationship between work experience and GMP practice have not been consistent across studies [5, 6, 14].

In Ethiopia, previous research has documented deficiencies in growth measurement, recording, growth chart interpretation, and counseling during GMP sessions [4, 21]. A study in Tigray also reported limited counseling based on growth curves [19]. Moreover, the conflict that began in Tigray in November 2020 severely affected the region′s health system, with many health facilities becoming partially or completely nonfunctional [22]. This disruption creates a need to understand the current status of GMP practice and the challenges faced by healthcare workers in delivering child growth monitoring and promotion services.

The significance of this study lies in its potential to provide evidence on GMP practice and associated factors among healthcare workers in public health facilities of central zone Tigray. The findings may support regional and woreda health authorities in identifying training needs, improving the availability of GMP equipment, strengthening supportive supervision, and allocating human and material resources. The study may also assist healthcare workers and nutrition program implementers in improving growth monitoring procedures and strengthening caregiver counseling. Furthermore, it can serve as a reference for future researchers investigating GMP implementation and child nutrition services in Ethiopia.

## 2. Methods

### 2.1. Study Area and Period

This study was conducted at public health facilities in the central zone of Tigray, Ethiopia. The central zone is one zone of the Tigray region. Its capital city is Aksum, which is 1165 km from Addis Ababa by car. According to the 2012 Health and Health‐Related Indicators published by MOH, Tigray has 41 hospitals, 226 health centers, and 743 health posts. Central zone has 212 health posts, 61 health centers, and 22 woredas [23]. From these, 10 woredas were randomly selected. Those are Keyh Tekli, Nadier, Aksum, Rural Adwa, Merebleke, Lalay Machew, Ahisea, Chila, Abergele Yechila, and Tanqua Milash. According to the 2023 report, the CZT health office had 795 healthcare workers who were directly involved in the GMP session. The data collection period was from November 27, 2023, to December 27, 2023.

### 2.2. Study Design

An institution‐based mixed study design was conducted.

### 2.3. Population

#### 2.3.1. Source Population

All healthcare workers who were providing GMP service at public health facilities of central zone Tigray.

#### 2.3.2. Study Population

All selected healthcare workers who were providing GMP service at the selected public health facilities of central zone Tigray during data collection.

### 2.4. Eligibility Criteria

#### 2.4.1. Inclusion Criteria

All healthcare workers working at under five (outpatient departments, outpatient therapeutic feeding program, and immunization) in the selected woreda health facilities during data collection were included.

#### 2.4.2. Exclusion Criteria

No exclusion criteria.

### 2.5. Sample Size Determination and Sampling Technique

#### 2.5.1. Sample Size Determination

The sample size for the quantitative study was calculated by using the single population proportion formula by considering the 95% confidence level, *z*‐value of 1.96, 5% margin of error, and 50.4% proportion of GMP practice, which was taken from a previous study done in north Gondar zone public health facilities [5].

Where *n* = the desirable sample size, *p* = 50.4*%*, Z_*α*/2_ = 1.96, and *d* = margin of error at (5%).

n=Zα/22p1−pd2,

*n* = (1.96)^2^∗0.504(1 − 0.504)/(0.05)^2^ = 384 by adding 10% nonresponse rate, the final sample size was 423.

The sample size for the second objective using significance associated factors that are taken from different literatures by considering confidence interval 95%, power 80%, and ratio 1:1 is as follows (Table 1).

**Table 1:** Sample size using significance associated factors by Epi Info Version 7.

Serial no.	Factors	Ratio	Power	CI	AOR	% of outcome in unexposed group	Sample size	Reference
1	Educational level	1:1	80%	95%	2.2	45%	228	[5]
2	Profession	1:1	80%	95%	0.42	52%	194	[5]
3	Knowledge	1:1	80%	95%	4.6	65.2%	106	[6]
4	Workload	1:1	80%	95%	3.02	68.18%	180	[6]
5	GMP equipment	1:1	80%	95%	2.75	35.7	142	[7]
6	Work experience	1:1	80%	95%	3.13	79.8%	260	[7]
7	Training	1:1	80%	95%	3.13	40.8%	116	[6]
8	Attitude	1:1	80%	95%	2.58	70.3	244	[6]

Sample size calculated using single population proportion formula is higher than the second objective using significance associated factors. Therefore, appropriate final sample size was 423.

Sample size for qualitative: Nine interviews were included in the qualitative study and were purposively selected based on their duration of service in GMP and their position.

#### 2.5.2. Sampling Technique and Procedure

There were 61 health centers and 212 health posts that have GMP service in the central zone of Tigray. For the quantitative part, there were 22 woredas in the zonal administration. From these, 10 woredas were selected by using the simple random sampling technique (lottery method). A total of 163 public health facilities (38 health centers and 125 health posts that provide GMP services) were included in the study. All healthcare workers who were directly involved in GMP at all health facilities of the selected woredas were included. For the qualitative part of the study, a purposive sampling technique (key informants for supervisors and medical directors and in‐depth interviews for healthcare workers) was used (Figure 1, Table 2).

**Figure 1:**
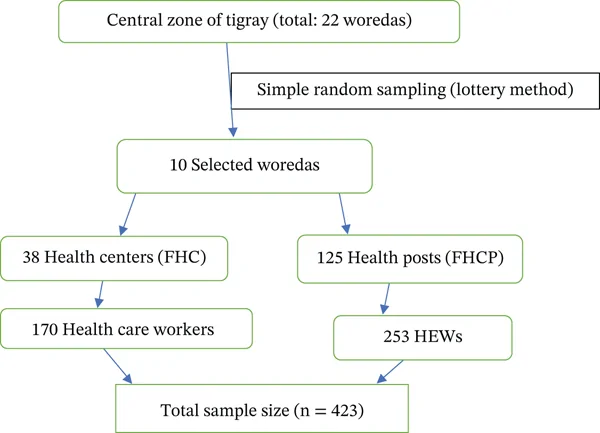
Diagrammatic presentation of sampling procedure for growth monitoring and practice and associated factors among healthcare workers in CZT Ethiopia, 2023/2024.

**Table 2:** Sampling distribution across selected woreda.

Selected woreda	Health centers	Health center staff (*n*)	Health post staff (*n*)	Woreda sample size (*N*)
Keyh Tekli	4	20	28	48
Nadier	4	20	26	46
Axum	2	10	28	38
Rural Adwa (Geter Adwa)	7	35	32	67
Merebleke	1	5	20	25
Lalay Machew	5	25	32	57
Ahisea	2	10	18	28
Abergele Yechila	3	15	29	44
Tanqua Milash	2	10	18	28
Chila	4	20	22	42
Total	34	170	253	423

*Note:* FHC = healthcare workers from health center; FHCP = health extension workers from health post; *N* = sample per woreda.

### 2.6. Study Variable

#### 2.6.1. Dependent Variables

GMP practice.

#### 2.6.2. Independent Variables

➢ Sociodemographic characteristics (educational status, profession, and work experience)
➢ Organizational factors (availability of GMP equipment, training, supportive supervision, and workload)
➢ Attitude
➢ Knowledge

### 2.7. Data Collection Tools and Procedure

Data collection tool was first prepared in English then translated to local language by language translator expert. Self‐administered questionnaire contains the following: sociodemographic factors, institutional related, knowledge related, attitude related, and checklist for the practice part. Observational checklist was used during GMP session to measure practice. Participants were provided with appropriate information about the study, then informed consent was obtained to assure their willingness to participate in the study. The participants were selected according to the eligibility criteria. Data were collected by data collectors using self‐administered structured questionnaire. It was collected by six BSc nurses and three health officer supervisors.

For the qualitative part, semistructured probing questions were used to collect the data from study participants. During in‐depth interviews and key informants, tape recording and field notes were used until the data got saturated. The duration of each interview was between 20 and 30 min. The qualitative data were collected by the principal investigator.

#### 2.7.1. Data Quality Assurance

The questionnaires were prepared in the English language, then it was translated to the Tigrigna language by an English expert. The questioner was adapted from previous literatures. A pretest was given to 5% of health workers from the total sample in Gororo health center and health post, then ambiguous questions were corrected accordingly. Cronbach alpha was computed to measure the internal consistency (reliability) between items in the scale for measuring knowledge, attitude, and practice towards GMP and was found to be 0.791, 0.812, and 0.909, respectively. The result showed that the item in each measurement scale were reliable and valid to use as a tool to collect the data.

Two days of training with practical exercise was given to data collectors and supervisors about the data collection process. Any problem faced during the data collection, discussion, and corrective measure was taken immediately by the data collectors, supervisor, and the principal investigator. The collected data were checked everyday by data collectors, supervisors, and the principal investigator for its completeness, accuracy, and consistency. Data were checked again for its completeness before data entry.

#### 2.7.2. Data Process, Data Analysis, and Interpretation

The collected data were checked for its completeness and consistency of the response. The data were then coded, edited, entered, and cleaned by Epi‐data manager Version 4.6 and then exported to SPSS Version 23 for analysis for the quantitative data. The data were manipulated using simple frequencies and cross‐tabulation before regression analysis. Frequency and cross tabulation were computed, and the results were summarized and presented by tables, charts, and texts. Percentage, frequency, and mean were calculated.

The binary logistic regression model was used to test the association between dependent and independent variables. All independent variables with *p* value < 0.25 in the bivariate logistic regression analysis were included in the multivariable logistic regression analysis. Consequently, multivariable logistic regression analysis was performed to identify the independent variables that showed significant association with the outcome variable. Magnitude of association was measured by using an odds ratio at 95% confidence interval. Statistical significance was declared at *p* < 0.05. Hosmer–Lemeshow test was used to check fitness model. The *p* value of the model fitness of test was 0.255, which is insignificant showing that the model is well‐fitted. Multicollinearity was checked by using variance inflation factor (VIF) and tolerance test. All variables had VIF of less than 3 (1.288–2.92) and tolerance test of greater than 0.2, showing no multicollinearity.

The tape‐recorded qualitative data were transcribed and translated to the English language by the PI. Then, the data were read, coded, displayed, reduced, and interpreted. Contents of in‐depth interview were grouped under selected themes based on questionnaire guides. Thematic analysis was done manually for the collected data. Finally, the results of the analysis were presented in the form of narratives.

### 2.8. Operational Definition

In this study, public health facilities indicate health centers and health posts.

#### 2.8.1. Practice

It was considered a good practice if health workers practiced at least 75% and poor practice if health worker practiced < 75% from the prepared checklist, respectively, by giving 1 for correct and 0 for incorrect practice from the 12 assessment checklists [6, 24].

#### 2.8.2. Workload

A healthcare worker who saw 25 children or more per day was regarded as busy and less than 25 children per day as ideal during GMP sessions [7].

#### 2.8.3. To Measure Attitude

The attitude of the health workers was measured by using 10 items of 5‐point Likert scale questions, which was scored out of 50. The questionnaires were given a score of 1–5. If a health worker answers ≥ 75%, it was consider as good attitude, whereas a score of < 75% was considered as poor attitude [5].

#### 2.8.4. Knowledge

To measure the knowledge of study participants about GMP, a score of 1 was given for a correct answer and 0 for an incorrect answer for the 14 prepared knowledgeable questionnaires. Poor knowledge was defined as a score of < 75%, and good knowledge was defined as ≥ 75% [5].

### 2.9. Ethical Consideration

After the study title was approved, an ethical clearance was obtained from the AUCH‐CSH research ethics review committee (IRB Number: IRB021/2023). A letter of permission was taken from the central zone health office to the selected woreda as per the preceding data collection. Participants were provided with appropriate information about the study. Informed consent and an audio record for the interviewee were secured prior to data collection, and the respondents were informed that they have the full right to withdraw or refuse at anytime from the process. Confidentiality of information given by each respondent was kept properly, and anonymity was explained clearly for the participant. Beneficence of the participants was maintained throughout the study.

## 3. Results

### 3.1. Sociodemographic Characteristics of Healthcare Workers

A total of 407 participants were included in the study with a response rate of 96.2%. Of those, 300 (73.7%) and 107 (26.3%) participants were female and male, respectively. The median age of the participant was 37 with an interquartile range of ±5. The majority of participants, 190 (46.7%), were nurses, followed by 175 (43%) health extension workers. Most of the health workers, 280 (68.8%), had 11 years and above of work experience. More than half, 231 (56.8%), of the participants′ workplaces were in health posts (Table 3).

**Table 3:** Sociodemographic characteristics of healthcare workers at public health facilities of CZT, northern Ethiopia, 2023/2024.

Sociodemographic variables	Category	Number	Percentage (%)
Sex	Male	107	26.3
	Female	300	73.7

Age	22–29	17	4.2
	30–39	289	71
	≥ 40	101	24.8
	Median (IQR)	37 ± 5	

Marital status	Single	51	12.5
	Married	328	80.6
	Widowed	28	6.9

Religion	Orthodox	398	97.8
	Others	9	2.2

Level of education	Certificate	42	10.3
	Diploma	232	57
	Degree	133	32.7

Profession	Health extension	175	43
	Nurse	190	46.7
	Health officer	42	10.3

Work experience	< 11 years	127	31.2
	≥ 11 years	280	68.8

Monthly income (palliative care and its associated factor)	4000–6000	39	9.6
	6001–8000	104	25.6
	≥ 8001	264	64.9

Workplace	Health post	231	56.8
	Health center	176	43.2

*Note:* In religion, others denote Muslim and Catholic.

### 3.2. Healthcare Worker′s Knowledge on GMP

Of the total participants, 386 (94.8%) and 320 (78.6%) knew the meaning of GMP and how to read the child growth chart, respectively. More than three‐fourths (331, 81.3%) knew the sensitive equipment of GMP and the correct order of GMP. More than half, 257 (63.1%), of the respondents knew what the plotted line between the lower and upper reference curves is. From the total study participants, 386 (90.4%) and 370 (90.9%) knew the minimum normal birth weight and maximum normal birth weight, respectively. The majority of the respondents, 351 (86.2%), knew about the time when an intervention is needed, and 376 (92.4%) healthcare workers knew that the frequency of GMP is every month. Of the total participants, 269 (66.1%) had good knowledge and 138 (33.9%) had poor knowledge, respectively (Table 4).

**Table 4:** Knowledge of healthcare workers towards GMP practice in central zone Tigray, northern Ethiopia from October 2023 to June 2024.

Variables	Category	Frequency	Percentage
Do you know the meaning of GMP?	Yes	386	94.8
	No	21	5.2
Do you know how to read the child growth chart?	Yes	320	78.6
	No	87	21.4
Can you interpret the child growth curve?	Yes	252	61.9
	No	155	38.1
Do you know the purpose of GMP?	Yes	379	93.1
	No	28	6.9
Do you know the sensitive equipment of GMP?	Yes	331	81.3
	No	76	18.7
Do you know the correct order of GMP?	Yes	331	81.3
	No	76	18.7
Do you know the plotted line above reference curve?	Yes	270	66.3
	No	137	33.7
Do you know the plotted line between lower and upper reference curve?	Yes	257	63.1
	No	150	36.9
Do you know the minimum normal birth weight?	Yes	368	90.4
	No	39	9.6
Do you know the maximum normal birth weight?	Yes	370	90.9
	No	37	9.1
Do you know when intervention is needed?	Yes	351	86.2
	No	56	13.8
Do you know the frequency of GMP per month?	Yes	376	92.4
	No	31	7.6
Do you know the recommended daily feeding of a 6‐ to 23‐month old breast feeding child?	Yes	301	74
	No	106	26
Do you know the reason for giving complimentary food at 6 months of age?	Yes	381	93.6
	No	26	6.4
Knowledge status	Good knowledge	269	66.1
	Poor knowledge	138	33.9

Abbreviation: GMP, growth monitoring promotion.

### 3.3. Attitudes of Healthcare Workers About GMP

About 196 (48.2%) of healthcare workers strongly agreed that GMP is necessary for every child. More than half (227 [55.8%]) of the healthcare workers had strongly agreed that GMP is effective in preventing childhood malnutrition. Nearly half (196 [48.2%]) of the participants strongly agreed that GMP is done not only for sick children. Only less than one‐third (119 [29.2%]) strongly agreed that the growth chart and growth card are used fully in the GMP session. More than half (250 [64.4%]) of the respondents strongly agreed that training is needed to enhance GMP. Of the total participants, 291 (71.5%) had a good attitude and 138 (33.9%) had a poor attitude, respectively (Table 5).

**Table 5:** Attitude towards GMP practice of healthcare workers at public health facilities of central zone Tigray, northern Ethiopia, 2023/2024 (*n* = 407).

Characteristics	Category	Frequency	Percentage (%)
GMP is necessary for every child.	Strongly disagree	19	4.7
	Disagree	24	5.9
	Neutral	13	3.2
	Agree	155	38.1
	Strongly agree	196	48.2

Weighing the child makes you happy.	Strongly disagree	16	3.9
	Disagree	35	8.6
	Neutral	15	3.7
	Agree	164	40.3
	Strongly agree	177	43.5

Process of GMP is not time consuming.	Strongly disagree	29	7.1
	Disagree	127	31.2
	Neutral	13	3.2
	Agree	125	30.7
	Strongly agree	113	27.8

GMP is effective in preventing childhood malnutrition.	Strongly disagree	13	3.2
	Disagree	17	4.2
	Neutral	19	2.2
	Agree	141	34.6
	Strongly agree	227	55.8

Mother/caregivers should be involve in GMP session.	Strongly disagree	7	1.7
	Disagree	29	7.1
	Neutral	18	4.4
	Agree	195	47.9
	Strongly agree	158	38.8

Process of GMP is not burdensome.	Strongly disagree	33	8.1
	Disagree	179	44.0
	Neutral	15	3.7
	Agree	108	26.5
	Strongly agree	72	17.7

GMP is done not only for sick children.	Strongly disagree	19	4.7
	Disagree	19	4.7
	Neutral	9	2.2
	Agree	196	48.2
	Strongly agree	164	40.3

Growth chart and growth card are use full in GMP session.	Strongly disagree	13	3.2
	Disagree	36	8.8
	Neutral	16	3.9
	Agree	223	54.8
	Strongly agree	119	29.2

Counseling mothers/caregivers and interventions make GMP complete.	Strongly disagree	11	2.7
	Disagree	22	5.4
	Neutral	9	2.2
	Agree	204	50.1
	Strongly agree	161	39.6

Training is needed to enhance GMP.	Strongly disagree	14	3.4
	Disagree	5	1.2
	Neutral	7	1.7
	Agree	131	32.2
	Strongly agree	250	61.4

Attitude status	Good attitude	291	71.5
	Poor attitude	116	28.5

Abbreviation: GMP, growth monitoring promotion.

### 3.4. Availability of GMP Equipment

From the total participants, about 268 (65.8%) reported that they have available equipment to do GMP practice, and 139 (35.2%) participants reported that they have no available equipment.

From the unavailable equipment, 117 (28.7%) and 150 (36.9%) of healthcare workers reported that there was a lack of a weight scale and a growth monitoring chart, respectively, in their health facilities (Figure 2).

**Figure 2:**
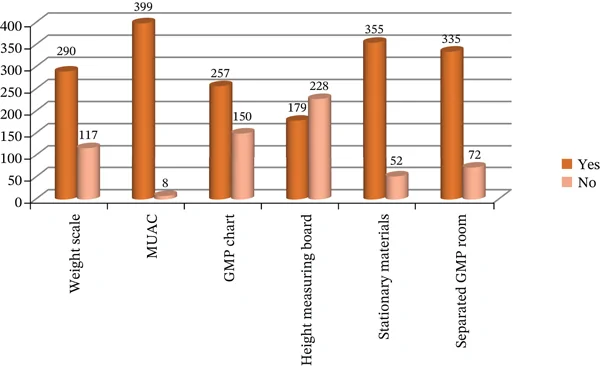
Availability of growth monitoring promotion equipment in central zone Tigray health facilities, 2023/2024.

### 3.5. Training of Health Workers and Supportive Supervision

Less than two‐thirds (245, 60.2%) of the participants replied that they had taken GMP training. The most common training types received by the respondents were malnutrition (162, 39.8%), IMNCI (41, 10.1%), immunization (28, 6.9%), and GMP (14, 3.4%). More than half, 156 (38%), of the training was provided by the regional health bureau, and 89 (21.9%) was given by the NGO.

Above half, 249 (61.2%), of the respondents had received supportive supervision. Of these, those who got supportive supervision (23.3%) were provided by the regional health bureau (Table 6).

**Table 6:** Percentage of training and supportive supervision of healthcare workers.

Variables	Category	Frequency	Percentage (%)
Training attended on GMP	Yes	245	60.2
	No	162	39.8
Training attended on (Option 1)	IMNCI	41	10.1
	Malnutrition	162	39.8
	Immunization	28	6.9
	GMP	14	3.4
Training provided by	RHB	156	38.3
	NGO	89	21.9
	Both (Option 2)	14	3.4
Option2	Malnutrition	15	3.7
	Immunization	12	2.9
	GMP	57	14
Option 3	Malnutrition	6	1.5
Number of training taken by HCWs	One times	148	36.4
	Greater than one	97	23.6
Supportive supervision	Yes	249	61.2
	No	158	38.8
Supportive supervision given by	HN	13	3.2
	RHB	95	23.3
	WHOF	68	16.7
	HEWs supervisors	66	16.2
	NGO	7	1.7
Option 2	RHB	3	0.7
	WHOF	6	1.5
	HES	38	9.3
Option 3	WHOF	2	0.5
	HEWS	2	0.5
	NGO	1	0.2

*Note:* Option 2 = participants who took training in addition to Option 1 (one from Option 1). Option 3 = participants who took training in addition to Options 1 and 2, Similarly, supportive supervision is given.

Abbreviations: GMP, growth monitoring and promotion; HEWS, health extension workers supervisor; HN, head nurse; NGO, nongovernmental organization; RHB, regional health bureau; WHOF, woreda health office.

### 3.6. Workload

Less than half, 190 (46.7%), of the participants reported that they had seen fewer than 25 children to follow the GMP session per day. More than half, 270 (59%), of the respondents took an average of 1–10 min for one child to assess GMP. Above half, 224 (55%), of the participants had another work in addition to GMP (Table 7).

**Table 7:** Workload for healthcare workers on GMP practice in central zone Tigray, northern Ethiopia, 2023/2024.

Variables		Frequency	Percentage
Number of children seen during GMP session per day	< 25 (ideal)	190	46.7
	≥ 25 (busy)	217	53.3
Do you have another work in addition to GMP?	Yes	270	59
	No	167	41
The average time in minute to assess GMP for one child	1–10	224	55
	> 10	183	45

Abbreviation: GMP, growth monitoring promotion.

### 3.7. Healthcare Workers′ GMP Practice

More than two‐thirds (291/71.5%) of the respondents took the child weight monthly while doing GMP in their health facilities. More than half of the respondents, 252 (61.9%), would undress/lighten the child before the child′s weight.

Of the 277 participants, 68.1% checked the accuracy of the weight scale, and above half, 258 (63.4%), adjusted the weight scale before weighing the child during GMP, respectively. Less than half, 194 (47.7%), more than half, 234 (57.5%), and more than three‐fourths, 360 (88.5%), of the participants interpret the growth curve, counsel mothers/caregivers based on the growth curve, and counsel mothers/caregivers when to introduce breast milk, solid, and semisolid for their child at the age of 6 months in addition to breast milk while doing GMP, respectively (Table 8). Of the total participants, 269 (66.1%) respondents had good knowledge, and 291 (71.5%) of the respondents had a good attitude (positive attitude) towards GMP (Table 9). The overall proportion of GMP practice among healthcare workers was 187 (45.9% [95% CI: 40.8–50.6]) (Figure 3).

**Table 8:** Healthcare workers GMP practice in central zone of Tigray, northern Ethiopia, 2023/2024.

Characteristics category		Frequency	Percentage
Weigh the child weight while doing GMP	Yes	291	71.5
	No	116	28.5
Check the accuracy of the weight scale while doing GMP	Yes	277	68.1
	No	130	31.9
Undress/light dress child while weighing the child weight	Yes	252	61.9
	No	155	38.1
Adjust the weight scale to zero before weighing the child weight	Yes	258	63.4
	No	149	36.6
Explain the growth curve to mother/caregiver while doing GMP	Yes	231	56.8
	No	176	43.2
Plot the growth curve on growth chart while doing GMP	Yes	100	24.6
	No	307	75.4
Interpret the growth curve while doing GMP practice	Yes	194	47.7
	No	213	52.3
Counsel mothers/caregivers based on growth curve when carrying out GMP	Yes	234	57.5
	No	173	42.5
Counsel mothers/caregivers when to introduce breast milk, solid, and semisolid for their child	Yes	360	88.5
	No	47	11.5
Mothers are part of GMP session	Yes	385	94.6
	No	22	5.4
Use growth chart	Yes	180	44.2
	No	227	55.8
Chart filled completely	Yes	214	52.6
	No	193	47.4

**Table 9:** Cross‐tab for the variables.

Variables	Category	GMP practice	
		Good practice	Poor practice
Sex	Male	83 (77.6%)	24 (22.4%)
	Female	104 (34.7%)	196 (65%)

Age	22–29	5 (29.4%)	12 (70.6%)
	30–40	144 (49.8%)	145 (50.2%)
	> 40	38 (37.6%)	63 (62.4%)

Marital status	Single	30 (58.8%)	21 (41.2%)
	Married	150 (45.7%)	178 (54.3%)
	Widowed	7 (25%)	21 (75%)

Religion	Orthodox	183 (46%)	215 (54%)
	Others	4 (44.44%)	5 (55.5%)

Level of education	Certificate	6 (14.3%)	36 (85.7%)
	Diploma	80 (34.5%)	152 (65.5%)
	Degree	101 (75.9%)	32 (24.1%)

Profession	Health ex	35 (20%)	140 (80%)
	Nurse	117 (61.6%)	73 (38.4%)
	Health off	35 (83.3%)	7 (16.7%)

Workplace	Health post	59 (25.5%)	172 (74.5%)
	Health center	128 (72.7%)	48 (27.3%)

Work experience on GMP	< 11 years	28 (22%)	99 (78%)
	≥ 11 years	159 (56.8%)	121 (43.2)

Income	4000–6000	5 (12.8%)	34 (87.2%)
	6001–8000	36 (34.6%)	68 (65.4%)
	≥ 8001	146 (55.3%)	118 (44.7%)

Available equipment	Yes	187 (69.8%)	81 (30.2%)
	No	0 (0.0%)	139 (100%)

Knowledge	Good	163 (60.4%)	106 (39.4%)
	Poor	24 (17.4%)	114 (82.6%)

Attitude	Good	159 (54.6%)	132 (45.4%)
	Poor	28 (24.1%)	88 (75.9%)

Training on GMP	Yes	157 (64.1%)	88 (35.9%)
	No	30 (18.5%)	132 (81.5%)

Supervision	Yes	153 (61.4%)	96 (38.6%)
	No	34 (21.5%)	124 (78.5%)

Workload	Ideal	144 (75.8%)	46 (24.2%)
	Busy	43 (19.8%)	174 (80.2%)

**Figure 3:**
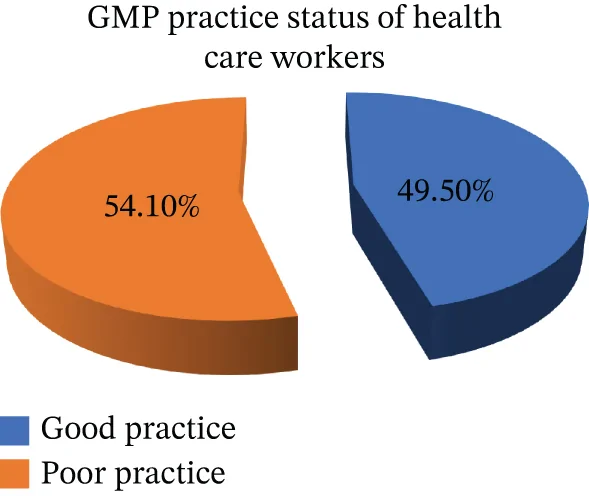
GMP practice status of healthcare workers.

### 3.8. Factors Associated With the GMP Practice of Healthcare Workers

A bivariate binary logistic regression analysis was performed to test the association between GMP practice and the independent variables (level of education, profession, work experience on GMP, knowledge on GMP, attitude towards GMP, training on GMP, supportive supervision on GMP, and workload of the healthcare workers towards GMP). All the independent variables show association with the outcome variable with a *p* value of < 0.25.

In the multivariable logistic regression analysis, educational status, profession, work experience knowledge, training on GMP, and the workload were significantly associated with GMP practice.

In the multivariable binary logistic regression, being a diploma holder healthcare worker was 74% less likely to practice GMP compared with a certificate (AOR = 0.260; 95% CI: 0.073, 0.923). Healthcare workers with a nurse profession were 2.489 times more likely to practice GMP compared with health extension workers (AOR = 2.489; 95% CI 1.211, 5.117). Health workers who had work experience of 11 or more years were 4.722 times more likely to be practicing GMP compared with health workers who had less than 11 years of work experience (AOR = 4.722; 95% CI: 1, 7.42).

The odds of GMP practice were 3.091 (AOR = 3.091, 95% CI: 1.499, 6.373) times more likely among health workers who had good knowledge compared with those who had poor knowledge. GMP practice was 3.404 times more likely among health workers who had received GMP training compared with those who had not received GMP training (AOR = 3.404; 95% CI: 1.386, 8.363). Health workers who saw fewer than 25 children per day were 6.651 times more likely to be practicing GMP compared with health workers who saw greater than or equal to 25 children per day (AOR = 6.651; 95% CI: 3.647, 12.129) (Table 10).

**Table 10:** Bivariate and multivariable logistic regression of growth monitoring promotion practice at public health facilities of central zone Tigray, northwest Ethiopia, 2023/2024 (*n* = 407).

Variables	Category	Practice of GMP		COR (95% CI)	*p*	AOR (95% CI)	*p*
		Good practice	Poor practice				
Level of education	Certificate	6 (14.3%)	36 (85.7%)	1		1	0.037∗
	Diploma (1)	80 (34.5%)	152 (65.5%)	3.158	0.013	0.260 (0.073–0.923)	
	Degree (2)	101 (75.9%)	32 (24.1%)	18.93 (7.3–49)	0.000	0.461 (0.106–2.003)	0.301
Profession	Health ex	35 (20%)	140 (80%)	1		1	0.013∗
	Nurse	117 (61.6%)	73 (38.4%)	6.4 (3.99–10.27)	0.000	2.489 (1.2–5.1)	
	Health off	35 (83.3%)	7 (16.7%)	20 (8.19–48.8)	0.000	3.84 (0.96–15.37)	0.057
Work experience on GMP	< 11 years	28 (22%)	99 (78%)	1		1	0.000∗
	≥ 11 years	159 (56.8%)	121 (43.2)	4.64 (2.87–7.5)	0.000	4.72 (2.3–9.6)	
Knowledge	Good	163 (60.6%)	106 (39.4%)	7.30 (4.41–12)	0.000	3.09 (1.49–6.37)	0.002∗
	Poor	24 (17.4%)	114 (82.6%)	1		1	
Attitude	Good	159 (54.6%)	132 (45.4%)	3.78 (2.33–6.14)	0.000	0.85 (0.387–1.88)	0.695
	Poor	28 (24.1%)	88 (75.9%)	1			
Training on GMP	Yes	157 (64.1%)	88 (35.9%)	7.85 (4.88–12.6)	0.000	3.4 (1.38–8.36)	0.008∗
	No	30 (18.5%)	132 (81.5%)	1		1	
Supervision	Yes	153 (61.4%)	96 (38.6%)	5.8 (3.67–9.1)	0.000	1.04 (0.417–2.59)	0.933
	No	34 (21.5%)	124 (78.5%)	1		1	
Workload	Ideal	144 (75.8%)	46 (24.2%)	12.667 (7.9–20.2)	0.000	6.65 (3.64–12.129)	0.000∗
	Busy	43 (19.8%)	174 (80.2%)	1		1	

Abbreviations: AOR, adjusted odds ratio; CI, confidence interval = the probability under the assumption; COR, crude odds ratio; GMP, growth monitoring promotion.

∗*p* value—significant.

### 3.9. Qualitative Result

Nine interviews were conducted (six in‐depth and three key informants) in the local language of Tigrigna. All the interviews were conducted at the workplace of the healthcare workers. The key informants were the supervisor and medical director. All the interviewees have five or more years of work experience in providing GMP service (See Table 11).

**Table 11:** Sociodemographic characteristics of the in‐depth interview and key informants.

Participants	Profession	Age	Sex	Level of education	Work experience	Position	Category
P1	Health officer	37	F	Degree	5 years	Medical director	KI
P2	Health officer	30	M	Degree	7 years	Supervisor	KI
P3	Health officer	36	F	Degree	12	Supervisor	KI
P4	Nurse	40	F	Diploma	10 years	—	IDI
P5	Health extension	38	F	Diploma	15 years	—	IDI
P6	Health extension	32	F	Diploma	11 years	—	IDI
P7	Nurse	31	F	Degree	8	—	IDI
P8	Nurse	32	M	Degree	8 years	—	IDI
P9	Health extension	39	F	Diploma	13 years	—	IDI

*Note:* “—” denotes no position.

Abbreviations: IDI, in‐depth interview; KI, key informants.

The main ideas that were interviewed with healthcare workers were sociodemography, definition of GMP, knowledge and counseling mothers/caregivers about child feeding, and barriers to GMP. The main findings are organized and presented below.

The majority of the respondents said that they gave counseling to mothers/caregivers. During the GMP session, they said that they gave more emphasis to infant breastfeeding and complementary feeding. In addition to that, they also focused on breast attachment and frequency of breastfeeding per day (10–12 times per day). A 40‐year‐old health extension worker with 10 years of work experience in GMP practice said, “During GMP practice, we gave counseling on the frequency of child feeding per day. We told the mothers/caregivers to provide only breast milk for their child until 6 months and then complementary feeding (semisolid and solid) at the age of 6 months.”

### 3.10. Activities Done in GMP Practice Area

The participants reported that GMP service was provided until 2 years. We are giving GMP service monthly on a selected day by measuring weight and comparing with WHO GMP chart to know the status of the child.

#### 3.10.1. Importance of GMP

The participants accepted that GMP had an advantage for children to have good nutritional status (to prevent stunting), physical and mental well‐being, and a productive society. A 37‐year‐old female medical director said that “GMP is used for children to have the fastest growth rate, to prevent malnutrition, to have good mental and physical well‐being, and to have a productive society.”

#### 3.10.2. Barriers of GMP Practice

Most interviewed participants recognize different barriers in the GMP program. The respondents explained that lack of GMP equipment, lack of awareness of mothers/caregivers, lack of knowledge, lack of motivated healthcare workers, lack of sufficient medical supply (Fafa and plump nuts, medications like albendazole, and vitamin A), lack of training, and understaffing (there are activities done in accordance with GMP) were the main barriers

### 3.11. Lack of GMP Equipment

Most of the interviewed participants said that lack of equipment was the main obstacle to giving GMP service, such as a weight scale, GMP chart, and GMP card.

A 33‐year‐old male supervisor with 7 years of work experience in GMP explains that “there is a lack of equipment such as a GMP chart, weight scale, and registration book.”

A 38‐year‐old female health extension worker with 15 years of work experience in GMP said that “there is a lack of equipment due to the genocidal war in Tigray, such as a weight scale, stationary materials like referral paper, GMP cards, and charts.”

### 3.12. Additional Works Besides GMP

Most interviewed participants identified different barriers, like additional activities in their health services and community.

A 32‐year‐old female nurse with 11 years of work experience working at a health post as a health extension coordinator reported that “healthcare workers had other additional work besides GMP, like immunization, maternal follow‐up, and giving awareness to the community about healthcare service.” She also said that “there was a shortage of healthcare workers, which is less than standard. The standard ratio of GMP per healthcare worker is 1 for 50 households. But I cover more than 1200 households.”

#### 3.12.1. Lack Training on GMP Practice

Most healthcare workers revealed that there was a shortage of training on GMP, which is important to update the knowledge and skills of healthcare workers.

A 38‐year‐old health extension worker with 15 years of work experience said that “there was a shortage of training related to GMP. Although we have gotten training one time on immunization and deworming by NGOs, it is not adequate.”

A 40‐year‐old diploma nurse said that “there was a lack of training related to GMP for healthcare workers for the last three years.”

#### 3.12.2. Knowledge of Healthcare Workers

Knowledge of healthcare workers has its own effect during GMP program implementation, as explained by most participants. A 31‐year‐old female health center working nurse said that “knowledge about GMP is mandatory to practice GMP effectively, and healthcare workers who had a good understanding of the growth chart are plotting and interpreting the graph easily.”

## 4. Discussions

This health facility‐based cross‐sectional study was conducted to assess GMP practices and associated factors among healthcare workers at public health facilities of the central zone, Tigray Region, northern Ethiopia. The finding of this study showed that the overall prevalence of GMP practice among healthcare workers was 45.9% (95% CI: 40.8–50.6). This result is in line with the prevalence of previous studies in Gondar, which is 50.4% [6]. However, it is lower than a study conducted in Tigray in 2018, South Africa in 2015, and Ghana in 2017, which are 53.6%, 60%, and 70%, respectively [14, 16, 19]. This variation is due the war in Tigray region of Ethiopia that in November 2020 has brought extremely large damage to the health system [22]. It may be also due to sociocultural difference [5]. This is supported by a qualitative study. A 36‐year‐old female supervisor said that “GMP practice was decreased from the previous due to war in Tigray that caused destruction and looting of the health facilities.”

However, this result was higher than that of the study done in Bahirdar and Banja, Northwest Ethiopia, which is 30.3% and 38.9% [6, 17]. This is due to study setting, assessment method used, sample size of the study, and sociodemographic factors [6]. Educational status, profession, work experience, knowledge, training, and workload were significantly associated with GMP practice among healthcare workers.

The odds of GMP practice among diploma‐holding healthcare workers were 74% less likely compared with the odds of GMP practice among certificate holders. However, a study conducted in Gondar shows that diploma healthcare workers are more likely than certificate holders. This contrast might be due to the study setting, the assessment method used, the sample size of the study, and sociodemographic factors.

The odds of GMP practice were 2.489 more likely among nurses compared with health extension workers. A similar finding was reported from the study done in Gondar. This is due to nurses having better knowledge about GMP than health extension workers [5]. This is supported by a qualitative study. A 36‐year‐old female supervisor said that “nurses are practicing GMP more than health extension workers because they are more familiar with how to plot graphs on the GMP chart, how to interpret the graph, and how to counsel mothers about child feeding.”

Healthcare workers with work experience of 11 years and above were 2.726 times more likely to practice GMP than their counterparts. This study is consistent with a previous study conducted in Tigray [19] and South Wollo zone, northeast Ethiopia [7]. Similarly, this study is in line with a study conducted in Bahirdar and Gondar [6, 7]. This is due to increased work experience enabling healthcare workers to have more chance to experience and perform procedures related to GMP practice [25] and in Ethiopia, GMP skills are mostly developed through experience and training [5]. This is supported by qualitative results. A 40‐year‐old male supervisor said that “during our supervision, more experienced healthcare workers practiced GMP since skill is gotten from life experience.”

However, a study conducted in South Africa shows that work experience inversely affects GMP practice in comparison with less experienced healthcare workers. This is due to the fact that senior healthcare workers see more patients per day, and this causes healthcare workers not to use growth charts regularly [14].

Healthcare workers who had good knowledge about GMP were 3.091 times more likely to practice GMP than those healthcare workers who had poor knowledge. This study had similar finding with study conducted in Nepal [3], South Africa [14], Bahirdar [6], and Tigray [19]. This due to knowledge being fundamental to any activities and having importance in practicing and implementing different healthcare procedures [6].

This is supported by a qualitative result: A 31‐year‐old female health center nurse said that “knowledge about GMP is mandatory to practice GMP effectively, and healthcare workers who had a good understanding of the growth chart are plotting and interpreting the graph easily.”

In this study, a positive association was found between health workers who attend training on GMP. Healthcare workers who received training on GMP were 3.404 times more likely to practice GMP than healthcare workers who had not received any training towards GMP. A similar finding was reported from a study conducted in Nepal, South Wollo Zone, Bahirdar, and Tigray [3, 6, 19, 7] showed a positive association and supported the result of this study. This might be also due to healthcare workers who receive training on GMP having to acquire technical skills, interpersonal skills, and good understanding to perform this job efficiently in their workplace [6]. The result of this was supported by a qualitative study. A 38‐year‐old health extension worker with 15 years of work experience said that “there was a shortage of training related to GMP. Although we have gotten training one time on immunization and deworming by NGOs, it is not adequate.” A 40‐year‐old diploma says that “there was a lack of training related to GMP for healthcare workers for the last 3 years.”

Healthcare workers who had seen less than 25 children per day were 6.65 times more likely to practice GMP compared with healthcare workers who saw 25 or more children per day. This study was in line with a study conducted by Ghana, South Africa, Bahirda, and Tigray [6, 14, 16, 19]. This might be due to busy healthcare workers being less concerned about practicing GMP. They are concerned for acutely sick children compared with healthy‐looking children [6]. The result of this *was* supported by the qualitative result as: A 32‐year‐old female nurse with 11 years of work experience working at a health post as a health extension coordinator reported that “healthcare workers had other additional work besides GMP, like immunization, maternal follow‐up, and giving awareness to the community about healthcare services.” “There is a shortage of healthcare workers, which is less than standard. The standard ratio of GMP per healthcare worker is 1 for 500 households. But I cover more than 1200 households.”

Unavailable equipment was one of the barriers to giving GMP service. A 33‐year‐old male supervisor with 7 years of work experience in GMP explains that “there is a lack of equipment such as GMP charts and registration books.” The barriers explained in this study were consistent with the barriers found in the study conducted in Gondar, Bahirdar, and Tigray [6, 7, 19]. This is due to looted and distracted the health facilities during the war in Tigray, northern Ethiopia [22].

A qualitative result showed that lack of awareness of mothers/caregivers, lack of equipment, and shortage of medication supply like albendazole and vitamin A are the common problems that were faced during GMP practice. This study was supported by a study conducted Tigray, Bahirdar, and Gondar [6, 7, 19]. This is due to the effect of war in Tigray, northern Ethiopia between Tigray regional government and Federal government [22]. A 31‐year‐old female nurse with 8 years of work experience said that “when we ask the responsible bodies to give as medical supply and equipment, their response were lack of budget.”

## 5. Conclusions and Recommendations

### 5.1. Conclusion

The overall proportion of GMP practice of healthcare workers of central zone Tigray public health facilities was low. This study had identified that level of education, profession, work experience, knowledge, training, and workload were factors associated with GMP practice among healthcare workers.

### 5.2. Recommendation

#### 5.2.1. Regional Health Bureau

The regional health bureau should strengthen the training on GMP, ensure adequate human resources, and retain more experienced healthcare workers at GMP practice site.

##### 5.2.1.1. For NGO

The nongovernmental organizations should work in collaboration with governmental health facilities to detect growth failure early and to prevent malnutrition by giving training on GMP.

##### 5.2.1.2. For Woreda and Zonal Health Bureau

The woreda and zonal health office should allocate the resources to health facilities based on their gap.

##### 5.2.1.3. For Healthcare Workers

The healthcare workers should update themselves instead of looking for higher officials training. They should give GMP service for the community.

##### 5.2.1.4. For Researchers

Researchers should conduct a study on mother′s attitude and other factors regarding GMP practice and meta‐analysis.

### 5.3. Strength and Limitations of the Study

#### 5.3.1. Strength

The strength of this study is using observational checklist to assess the level of GMP practice of healthcare workers. Both quantitative and qualitative data were used to support the result and to explore factors that are not addressed by quantitative method, which increases the validity of the data. Audio recording equipment was used during the in‐depth interview.

#### 5.3.2. Limitations

Although the observational checklist had advantages in improving the reliability of the study, its use might have introduced observational bias. Furthermore, the possibility of a Hawthorne effect could not be excluded, as healthcare workers might have changed their behavior because they knew they were being observed. The study included only healthcare workers and did not incorporate the perspectives of mothers and caregivers, which limited a comprehensive understanding of GMP services. The limited number of qualitative participants might also have reduced the diversity of perspectives captured. In addition, the measurement tools and classification criteria might not have fully captured all dimensions of GMP practice. Finally, the findings might have limited generalizability to healthcare workers and health facilities outside the study area.

**Nomenclature**
CZT: central zone of Tigray
ETB: Ethiopian birr
GMP: growth monitoring promotion
MOH: Ministry of Health
MUAC: mid‐upper arm circumference
NGO: nongovernmental organization
PI: principal investigator
SPSS: statistical package for social sciences
WHO: World Health Organization

## Author Contributions

G.H.B. conceived and designed the study, analyzed the data, and wrote the manuscript. G.K.A. was involved in data analysis, drafting of the manuscript, and advising the whole research paper; was involved in the interpretation of the data; and contributed to manuscript preparation.

## Funding

No funding was received for this manuscript.

## Disclosure

All authors have read and approved the final version of the manuscript.

## Ethics Statement

After the study title was approved, an ethical clearance was obtained from AUCH‐CSH research ethic review committee (IRB Number: IRB021/2023). A letter of permission was taken from central zone health office to the selected woreda as preceding data collection. Participants were provided with appropriate information about the study.

## Consent

Participants were provided with appropriate information about the study. Informed consent and audio record for the interviewee were secured prior to data collection, and the respondents were informed that they had full right to withdraw or refuse at anytime from the process. Confidentiality of information given by each respondent was kept properly and anonymity was explained clearly for participants. Beneficence of the participants was maintained throughout the study.

## Conflicts of Interest

The authors declare no conflicts of interest.

## Data Availability

The data used to support the findings of this study are included in the article.
